# A Recombinant Multiepitope Protein for Hepatitis B Diagnosis

**DOI:** 10.1155/2013/148317

**Published:** 2013-11-05

**Authors:** Marilen Queiroz de Souza, Alexsandro Sobreira Galdino, José Carlos dos Santos, Marcus Vinicius Soares, Yanna C. de Nóbrega, Alice da Cunha Morales Álvares, Sonia Maria de Freitas, Fernando Araripe Gonçalves Torres, Maria Sueli Soares Felipe

**Affiliations:** ^1^Laboratório de Biologia Molecular, Universidade de Brasília, 70910-900 Brasília, DF, Brazil; ^2^Laboratório de Biotecnologia de Microrganismos, Universidade Federal de São João Del-Rei, 35501-296 Divinópolis, MG, Brazil; ^3^Laboratório de Imunopatologia, Departamento de Patologia Molecular, Universidade de Brasília, 70910-900 Brasília, DF, Brazil; ^4^Laboratório de Biofísica, Instituto de Ciências Biológicas, Universidade de Brasília, 70910-900 Brasília, DF, Brazil; ^5^Pós-Graduação em Ciências Genômicas e Biotecnologia, Universidade Católica de Brasília, 70910-900 Brasília, DF, Brazil

## Abstract

Hepatitis B is a liver inflammation caused by hepatitis B virus (HBV) and can be diagnosed in clinical stage by hepatitis B core antibody from IgM class (anti-HBcIgM). Hepatitis B core antibody from IgG class (Anti-HBcIgG) appears quickly after IgM, reaching high titers in chronic hepatitis, and remains even after cure. Since hepatitis B core antibody (anti-HBc) is the first antibody identified and sometimes the only marker detected during the course of infection, it can be used both to indicate HBV acute infection (anti-HBc-IgM) and to identify individuals who have come into contact with the virus (anti-HBc-IgG). In this work we propose a recombinant hepatitis B core multiepitope antigen (rMEHB) to be used for diagnosis of hepatitis B. For this purpose, a synthetic gene coding for rMEHB was designed and cloned into vector pET21a with a 6xHis tag at the C-terminal. Time course induction in *E. coli* showed an induced protein with an apparent molecular mass of ~21 kDa. Protein purification was performed by a single step with affinity chromatography Ni-NTA. Circular dichroism spectroscopy indicated rMEHB as a thermal stable protein at pH 7.0 and 8.0. In these conditions rMEHB was successfully used to perform an enzyme linked immuno sorbent assay (ELISA) with positive and negative sera.

## 1. Introduction

Infections caused by hepatitis B virus (HBV) are a public health problem that concerns the entire world [1]. About a third of the world's population has already had contact with the virus during their lifetime. The World Health Organization (WHO) has estimated that there are more than 2 billion HBV infected individuals and about 378 million chronic carriers worldwide. Approximately 4.5 million new cases of HBV infection occur per year and a quarter progresses to liver disease [2]. HBV infection has a wide spectrum of liver diseases ranging from acute or fulminant hepatitis, chronic hepatitis, and cirrhosis to hepatocellular carcinoma (HCC) [3]. Infection occurs very often in early childhood when it is asymptomatic and often leads to the chronic carrier state [4]. In low endemic region like in the Central Asian republics, Southeast Asia, Subsaharan Africa, and the Amazon basin, the HBV carrier rate is over 8% [2], whereas in low endemic regions like the United States, Northern Europe, Australia, and parts of South America, it is less than 2% [2]. 

The HBV genome is a partially double-stranded DNA comprising about 3,200 nucleotides [5]. The genome is compact and contains sequences for four overlapping open reading frames (ORF) that encode structural and nonstructural proteins of the virus [6]. The first, ORF P, codes for a terminal protein on the minus strand as well as viral polymerase. ORF C codes for nucleocapsid structural protein as well as the hepatitis e antigen (HBeAg), which is responsible for immunomodulation and replication inhibition function. ORF S/pre-S codes for viral surface glycoproteins (HBsAg) that bind to cell receptors and facilitate viral entry [7]. HBV has a polyhedral structure composed of identical subunits of 21 kDa and it is serologically defined as HBcAg which has been proposed to be related to HBeAg, a second HBV-induced antigen based on the fact that HBcAg can be converted into HBeAg after proteolysis [8].

The virus interferes with the function of the liver while replicating in hepatocytes. As a result of pathological damage, the liver becomes inflamed [4]. Currently, four major serotypes and nine minor subtypes have been serologically identified [3]. The complete DNA sequencing of HBV isolates worldwide have led to the identification of eight genotypes (A to H) and a number of subgenotypes, showing different ethno/geographic distribution [3, 9]. Genotype A has been reported in Northern Europe, North and South Americas, India, and Central Africa, while isolates belonging to genotypes B and C have been observed in Southeast Asia and the Far East. Genotype D has a worldwide distribution and predominates in the Mediterranean and Middle East regions. Genotype E and F are prevalent in West Africa and in Amerindian populations, respectively. Genotype G has been identified in Europe, Mexico, and the USA, and genotype H has been found in Central America [10–12]. In Brazil, all genotypes can be found being genotypes A the most prevalent [13]. Despite a safe and effective vaccine is being available for more than two decades, HBV infection is still regarded as a global health problem [3].

The diagnosis of an infection by HBV can be carried out by molecular tests (quantitative and qualitative search of HBV DNA) and by serological tests. In this case, HBsAg and HBeAg, and anti-HBsAg, anti-HBeAg, and anti-HBcAg antibodies are identified in the serum during infection [14]. These antigens and antibodies appear and disappear in the serum according to the evolutionary phase of the illness [15]. During the incubation period, a few days after the appearance of antigen HBsAg, anti-HBc antibodies are detected [16]. In the initial phase, the IgM class of antibodies (anti-HBc-IgM) is predominated, remaining up to three months after the beginning of the clinical signs. During infection, anti-HBc from the IgG class (anti-HBc-IgG) presents ever-growing titers and remains detectable during its lifetime [2]. Therefore, while anti-HBc-IgM represents an important contribution to the diagnosis during the acute phase of the infection, anti-HBc-IgG is an important clinical and epidemiological marker for this infection. Patients that remain positive for many years towards anti-HBc markers run the risk of transmitting the illness on rare occasions (when donating organs or tissues) or reactivating infection by HBV when immunosuppressed [7]. During routine clinical practice, total anti-HBc serological tests are used in order to detect IgM and IgG antibodies [2]. Although the HBsAg is the most abundant protein used as diagnostic marker of hepatitis B infection [17], the test for anti-HBs can be ambiguous either by referring to the patients who came in contact with the HBV, or simply patients vaccinated but never came in contact with HBV. Furthermore, HBsAg is very heterogeneous [17] and a diagnostic test using multieptope protein from HBsAg would have to consider all immunodominant epitopes, which would thus result in a very large protein. On the other hand, because the coding region for HBcAgis conserved in the different HBV genotypes [15], the use of this region for diagnostic purposes could be more reliable.

There is a great need to develop highly efficient and inexpensive diagnostic methods, which should also be sensitive and specific. The development of recombinant proteins containing a high density of conserved epitopes has been a rational strategy for antibody recognition in diagnostic [18–24]. Multiepitope proteins have shown a great capacity to expose a wide range of epitopes with great efficiency, thus permitting the increase of the sensitivity of the test [18–24]. Several studies have been published on multiepitopes proteins for use successfully in diagnostic [18–24]. However, to date, no studies on the use of multiepitope proteins for hepatitis B diagnosis have been published. This prompted us to develop a recombinant multiepitope antigenic protein from the nucleocapsid of HBV (HBcAg) that could be useful for hepatitis B diagnosis.

## 2. Material and Methods

### 2.1. Design and Cloning of the Synthetic Gene

The gene coding for the recombinant hepatitis B multiepitope protein (rMEHB) was designed after selecting four conserved epitopes from the HBV virus (Table 1) and taking into account the *E. coli* codon usage. A609 pb*Nde*I/*Xho*I fragment was custom synthesized (Epoch Biosciences) and cloned into pET21a resulting in plasmid pET (rMEHB). The synthetic gene contained 3 copies of a stretch of the following epitope: epitopes 1 and 2 were separated by glycine and serine (GSGSG) flexible linkers, and epitopes 3 and 4 were present *in tandem*. The gene also contained six histidine residues (His-tag) at the C-terminal to allow protein detection and purification. The rMEHB consists of epitope 1 (CWGELMNLATWVGSNLEDPASRE) epitope 2 (CLTFGRETVLEY) and fused epitopes 3 and 4 (TPPAYRPPNAPILSTLPE). All epitopes were separated by the refereed flexible link. The structure showed above was repeated for three times originating a 21 kDa recombinant protein.

### 2.2. Expression and Purification of rMHEB

*E. coli *BL21 (*λ*DE3) pLysS cells were transformed with the pET (rMEHB) and a single colony was inoculated in Luria-Bertani (LB) medium and incubated at 37°C with shaking overnight. The culture was grown until optical density (OD_600 nm_) until 0.6 when 1 mM of IPTG was added. Bacteria from induced culture were centrifuged at 6000 ×g for 30 min at 4°C and the resulting pellet was stored at −80°C. The purification strategy involved addition of lysis buffer (8 M urea, 50 mM NaH_2_PO_4_, 300 mM NaCl, 10 mM imidazole, and pH 8.0) to the pellet following incubation at 4°C overnight. The system was then sonicated and 500 *μ*L Ni-NTA resin (Amersham Bioscience) was added to the supernatant for batch purification. For purification the resin was washed (8 M urea, 50 mM NaH_2_PO_4_, 300 mM NaCl, 20 mM imidazole, and pH 8.0) and proteins eluted with 8 M urea, 50 mM NaH_2_PO_4_, 300 mM NaCl, and 500 mM imidazole (pH 8.0).

### 2.3. Electrophoresis and Western Blotting

Protein samples were separated on 12% SDS-PAGE and electroblotted onto polyvinylidenefluoride (PVDF) membrane. The membrane was blocked with 5% milk in PBST (7 mM Na_2_HPO_4_, 137 mM NaCl, pH 7.4, and 1% Tween 20) for 2 h at room temperature. After 3 washes with PBS, the membrane was then incubated for 2 h at room temperature with monoclonal mouse anti-poliHis AP (Alkaline Phosphatase, Sigma), diluted 1 : 1000 in PBS. After three washes with PBST the specific protein band was visualized by the nitroblue tetrazolium/5-bromo-4chloro-3′-indolylphosphate (NBT/BCIP) detection method. 

### 2.4. Circular Dichroism Spectroscopy

Circular dichroism (CD) assays were carried out using Jasco J-815 spectropolarimeter (Jasco, Tokyo, Japan) equipped with a Peltier-type temperature controller. Far-UV spectra (260 to 195 nm) of rMEHB (0.08 mg*·*mL^−1^) were analyzed in 2 mM MOPS buffer pH 7.0 and 8.0 at 25°C using 0.2 cm path length quartz cuvette. Four consecutive measurements were accumulated and the mean spectra were recorded. The observed ellipticities were converted into molar ellipticity ([*θ*]) based on molecular mass per residue of 115 Da. Thermal denaturation assays of rMEHB were performed, raising the temperature from 25°C to 95°C, and monitored by dichroic signal at 200 nm. The temperature and pH dependence on secondary structure content were estimated from far-UV CD curve adjustments, using the network secondary structure estimator from concentration-independent method (http://perry.freeshell.org/raussens.html) [25]. In order to support the secondary structure prediction, the rMEHB amino acid sequence was submitted to network protein sequence analysis program [26].

### 2.5. Activity Test of rMEHB

The immune enzymatic assay was used to detect specific binding of the recombinant protein rMEHB to anti-HBc. A commercial HBV diagnostic kit (ETI-EBK PLUS No 140—Diasorin) was used. In this kit, the provided antigen was replaced by rMEHB, and a monoclonal anti-HBc was used. For each well different concentration 50 ng, 500 ng, 1 *μ*g, 10 *μ*g, and 15 *μ*g of rMEHB were added in triplicate. After incubation at 37°C for 2 h, the wells were washed with PBST. Then, 100 *μ*L of secondary antibody (monoclonal mouse anti-poliHis AP—Sigma) were added. After incubation at 37°C for 2 h, the plate surface was again washed with PBST. After this procedure, *p*-nitrophenyl phosphate (pNPP) was added as substrate for alkaline phosphatase following detection at 405 nm. The commercial kit was based on a competitive test in which the antibody present on the plate surface competes with the antibody present in serum of patients infected by the antigen. For this, the positive and negative sera of the commercial kit were used as controls.

## 3. Results and Discussion

### 3.1. Design of the Hepatitis B Multiepitope Protein (rMEHB)

The use of multiple native or recombinant proteins in diagnosis has an important impact on the price of immune-trial kits. Alternatively, the use of multiepitope proteins produced in *E. coli* and purified by a single step procedure allows the design of less expensive kits which can be used routinely in laboratory tests [18]. In addition, the rationally designed recombinant proteins presenting high epitope density may provide increased sensitivity and specificity [18–24].

**Figure 1:**
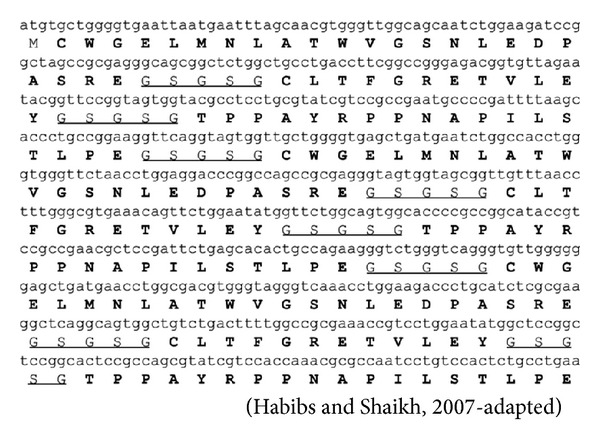
Nucleotide and predicted amino acid sequences of rMEHB.

The multiepitope protein developed in this work consists of a single polypeptide chain containing conserved epitopes which are prevalent worldwide, particularly in Brazil. These epitopes correspond to viral regions that are known to be capable of efficiently recognizing specific HBc antibodies in infected individuals. The epitopes used to design the antigenic protein are derived from structural core regions and were selected based on four criteria: (i) immunodominance, (ii) specificity towards anti-HBc antibodies, (iii) sequential construction of the epitopes separated by specific linker, and (iv) phylogenetic conservation of the majority of the HBV genotypes. Four epitopes from the core region were selected for rMEHB (Table 1) and were separated by glycine and serine flexible linkers. In order to increase epitope density the four epitope cluster was repeated three times resulting in a peptide of ~21 kDa which was called rMEHBV (Figure 1).

### 3.2. Expression and Purification of rMEHB

The use of recombinant multiepitope proteins over expressed in *E. coli *can be used in diagnostic kits for hepatitis B with several advantages: lower costs, facilitated manipulation, and elimination of problems concerning concentration of different peptides in the kits [18–24].

**Figure 2:**
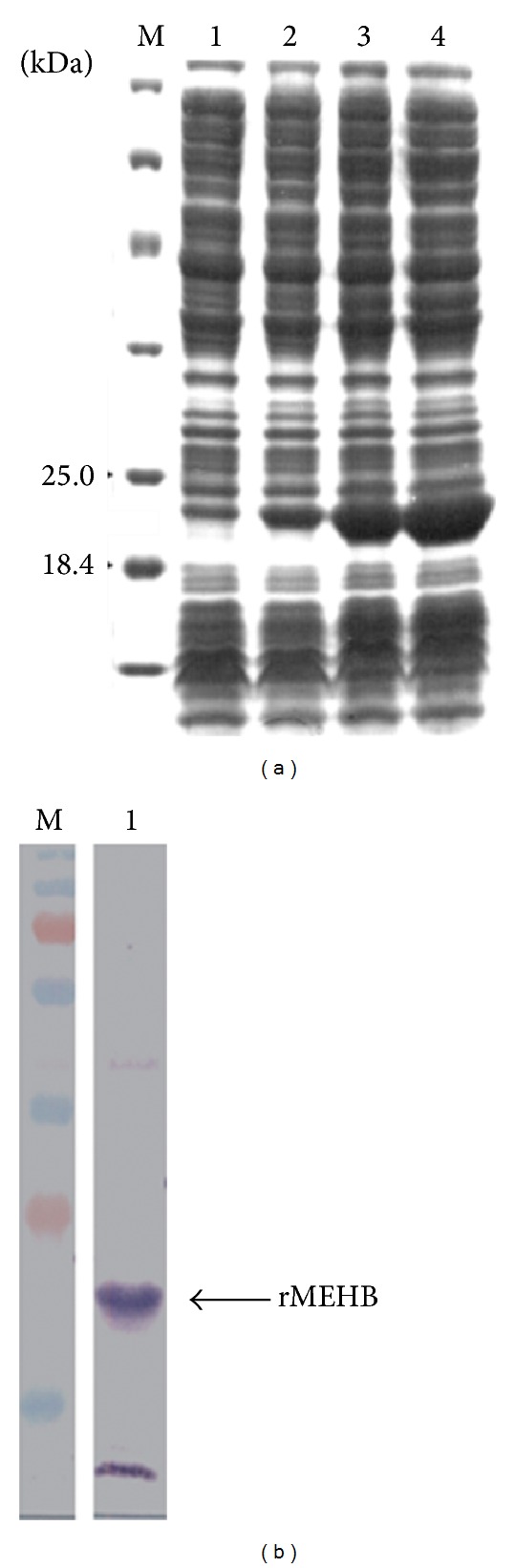
Time course rMEHB expression in *E. coli *BL21 (*λ*DE3) and analysis by SDS-PAGE12%. (a) Lane M, unstained protein molecular mass marker (Fermentas Life Sciences); Lane 1, uninduced control; Lanes 2–4, after 0.5, 1.5, and 2.5 hours of induction, respectively. (b) Western blotting analysis of the purified rMEHB. The monoclonal antibody, antipolyhistidine conjugated alkaline phosphatase, was diluted 1 : 1000 in PBS.

**Figure 3:**
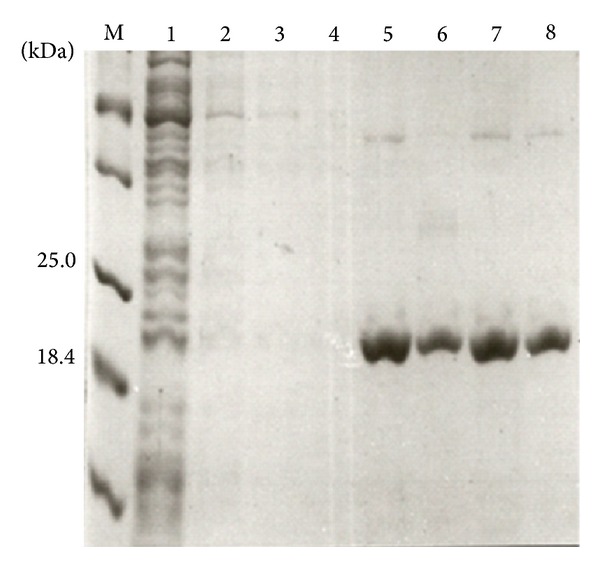
Analysis of rMEHBby SDS-PAGE 12% after purification procedure. Fractions were collected after Ni-NTA chromatography. Lane M, molecular mass markers (Weight Standard, Broad Range, BioRad). Lane 1, flow-through; Lanes 2–4, wash steps; Lanes 5–8 purified rMEHB after elution with 500 mM imidazole.

 As shown in Figure 2(a), induction of protein expression in *E. coli* resulted in the appearance of a ~21 kDa species (consisted with the expected size of rMEHV) which was confirmed by Western blotting to have a his tag (Figure 2(b)). The expressed rMEHB formed inclusion bodies that were disrupt and solubilized by using 6 M urea. Fractions collected during different steps of the purification were analyzed by SDS-PAGE (Figure 3). Three steps of washing remove most protein contaminants. Elution of the bound proteins using 500 mM imidazole resulted in highly purified rMEHB rendering 1.5 mg from 25 mL of soluble protein obtained after cells lysis (Figure 3, lanes 6–9).

### 3.3. Circular Dichroism Spectroscopy Analysis

**Figure 4:**
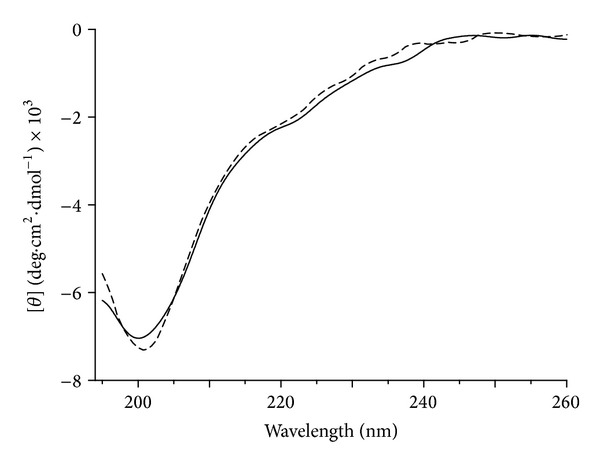
Far-UV CD spectra of rMEHB (0.08 mg·mL^−1^) in 2 mM MOPS buffer at 25°C. Solid and dashed lines indicate the CD spectra of MEHB at pH 7.0 and 8.0, respectively.

Circular dichroism is a useful spectroscopic tool to predict secondary structures of proteins and to correlate conformation properties of proteins dependent on environmental conditions [32, 33]. This technique was performed with the purpose of investigating the structural stability and secondary structure arrangement of the recombinant protein rMEHB at different pHs and temperatures. CD spectra of rMEHB at pH 7.0 and pH 8.0 25°C were similar to each other and displayed a negative band at 200 nm, typically being an unordered structure pattern (Figure 4). The secondary structure content of rMEHB estimated from spectra according to the Raussens et al. method [25] shows predominance of unordered and *β*-sheet structures in both pH 7.0 and 8.0 at 25°C (Table 2). These results are consistent with those obtained according to secondary structure prediction by network protein sequence analysis program in which 43.9% *β*-strand and 44.4% unordered structure were predicted.

**Figure 5:**
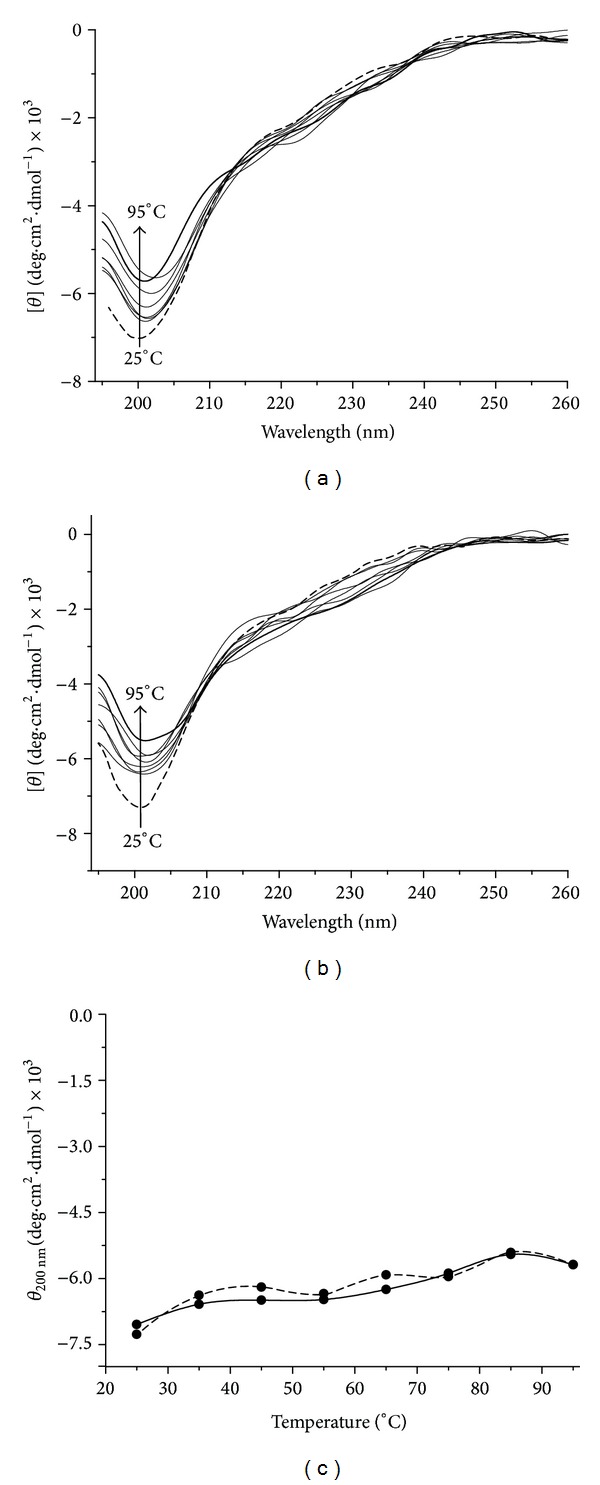
Heat-induced unfolding of rMEHB by circular dichroism (CD). (a) Far UV CD spectra in 2 mM MOPS buffer pH 7.0 and (b) pH 8.0, as a function of temperature. Molar ellipticities [*θ*] were measured from 260 to 195 nm at temperature rising from 25°C to 95°C. The arrows indicate decreased dichroic signal at 200 nm with increased temperature. (c) Temperature induced unfolding curves of rMEHB in 2 mM MOPS buffer pH 7.0 (––) and pH 8.0 (—) monitored at 200 nm.

Thermal stability of recombinant protein at pH 7.0 and 8.0 was monitored by gradual decrease of the dichroic signal at 200 nm (Figures 5(a) and 5(b)). As shown in Figure 5(c), no pattern of protein denaturation could be verified, as evidenced by no significant changes in the molar ellipticity as a function of the temperature. However, the dichroic signal at 200 nm decreasing from about −7,300 to −6,300 degree·cm^2^·dmol^−1^ (Figure 5(c)) indicates that the secondary structure alteration throughout the temperature range of 25 to 95°C is compatible with low conformational changes in the protein. These results indicate that rMEHB is a thermal stable protein at pH 7.0 and 8.0 upon raising the temperature from 25°C to 95°C, since no denaturing process was observed in these conditions.

 The high stability of protein at both pHs can be justified, in part, by the amino acid composition. Overall, the most abundant amino acid residues composing the rMEHB are serine plus threonine (19.5%) and glycine plus proline (26.3%). These amino acids provide a high content of hydrogen bonding (hydrogen bond-forming residues) and polypeptide folded in globular protein (turn and loop forming residues), respectively, favoring a more compact state resulting in the stabilization of the protein even at 95°C. Furthermore, it can be also justified by the instability index (II) of 36.14, a physical-chemical parameter used for characterization of protein stability [25], predicted for amino acids sequence (http://web.expasy.org/protparam/) that classifies rMEHB as a stable protein.

 Analysis of these structural parameters is very important, considering that rMEHB, a recombinant multiepitope protein, was expressed in order to preserve its tridimensional structure and the exposed regions containing defined epitopes for diagnostic purposes. Additionally, the stability of rMEHB is of fundamental importance in order to facilitate its transport for hepatitis B diagnosis. Finally, besides the high thermal stability, the results revealed the similar folding pattern of rMEHB under neutral and alkaline conditions and confirmed that recombinant protein can be used under these conditions for hepatitis B diagnosis. 

### 3.4. Activity Test of rMEHB

**Figure 6:**
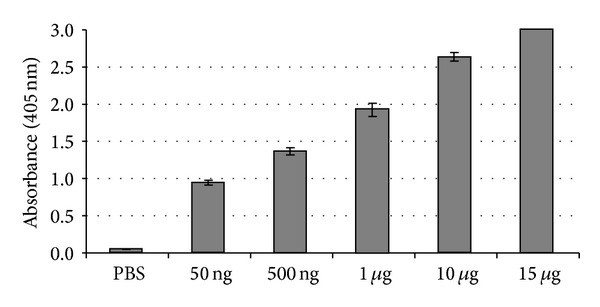
Enzyme linked immuno sorbent assay (ELISA) using rMEHB and monoclonal antibody Anti-HB, present on the plate surface of a commercial kit for hepatitis B diagnosis. Several concentrations of rMEHB were used as following: 50 ng, 500 ng, 1 *μ*g, 10 *μ*g, and 15 *μ*g. PBS was used as negative control. Antipolyhistidine conjugated alkaline phosphatase was the second antibody and the substrate was pNPP.

**Figure 7:**
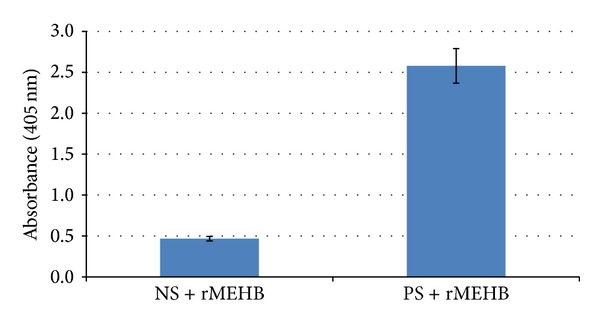
Immunological assay of rMEHB. The commercial antigen was replaced by rMEHB for the competitive test. NS: commercial negative control serum. PS: commercial positive control serum. Antipolyhistidine conjugated alkaline phosphatase was the second antibody and the substrate was pNPP.

In order to compare antigenic activity of the recombinant protein, a commercial diagnostic kit containing a monoclonal anti-HBc capable of recognizing anti-HBc was used.  Figure 6 shows that rMEHB in different concentrations was recognizes by anti-HBc antibodies. Also, the ability of rMEHB to be used as a diagnostic test for hepatitis B was confirmed by immune assay, replacing a commercial antigen by rMEHB (Figure 7). The recombinant antigen HBcAg binds to the anti-HBc antibodies coated on the board or binds to antibodies present in the sample. For this test, the commercial antigen was also used as a control (data not shown), and it was observed that the recombinant antigen rMEHB responds as well as the commercial antigen. 

## 4. Conclusions

The rMEHB could be used as input for hepatitis B diagnosis ensuring the use of healthy blood. The high density and the solvent-exposed of the conserved epitopes in the recombinant protein could contribute to a high degree of specificity and sensitivity, although not yet determined. The CD data showed that rMEHB is a stable protein mainly folded as unordered and *β*-strand structures. This structural feature probably favors the epitopes exposure and consequently the epitope-antibody recognition and binding processes. Furthermore, the high level of expression of rMEHB in *E. coli* and its single-step affinity purification make this approach an inexpensive process to obtain this protein.

## Figures and Tables

**Table 1:** HBcAg epitopes selected to compose the recombinant protein.

Epitope	Residues	References
(1) CWGELMNLATWVGSNLEDPASRE	61–83	Sallberg et al., 1994 [27]; Salfeld et al., 1989 [8] and Thermet et al., 2004 [28].
(2) CLTFGRETVLEY	107–118	Collucci et al., 1988 [29].
(3) TPPAYR	128–133	Sallberg et al., 1991 [30].
(4) PPNAPILSTLPE	134–145	Takahashi et al., 2001 [31].

**Table 2:** Secondary structure content of rMEHB calculated from far-UV (260–195 nm) CD curves adjustments in 2 mM MOPS pH 7.0 and 8.0 using the network secondary structure estimator from concentration independent Raussens et al. method (http://perry.freeshell.org/raussens.html) [25].

Secondary structure (%)	pH 7.0		pH 8.0	
	25°C	95°C	25°C	95°C
*α*-helix	7.5	5.3	8.0	7.4
*β*-sheet	32.7	36.7	33.1	33.1
*β*-turn	12.5	14.5	12.5	12.9
Random-coil	40.9	40.5	40.8	40.9
